# Review of the Proceedings of Last Meeting

**Published:** 1846-09

**Authors:** 


*Review of the Proceedings of Last Meeting.*—The American Society of Dental Surgeons having just closed another annual session, and much important business having come up for action, we have thought that a brief review of its proceedings might not be uninteresting to the readers of the Journal, eapecially to those who were not present at this meeting. This session was principally occupied in carrying out measures introduced at the meeting of last year, viz. amending and revising the constitution and by-laws; and carrying out the resolutions adopted at the last session, pertaining to the subject of amalgams.

One of the principal objects of the proposed amendments was, to protect the Soctety more effectually against the admission of unworthy candidates. As it seemed desirable to make some change in this particular before another election of members took place, and as the proposed candidates, hence, were not ballotted for, a full explanation is due those whose names had been duly presented to the Society as candidates for membership.

By consulting the record of the annual session of 1845, it will be perceived that not a single candidate was admitted to membership; and the record of the present year shows that only *one* was admitted this year.

This record, unexplained, would indicate, either that there were no candidates proposed, or that all who were proposed were unworthy the full confidence of the society; whereas, neither was in fact the case, for at each of these sessions there were many most unexceptionable names presented.

But why were no more elected? It had become more and more apparent, ever since the adoption of the original constitution, that sufficient guards were not thrown around the Society, in respect to the admission of members, and from facts developed at the session of 1845, the Society felt constrained to stop all further ingress, till some change should be made in this feature of the constitution. Hence it was "resolved" that no election of members should take place during that session; nor till after some amendments were adopted in respect to this point. It was at the same time provided, that each candidate should be notified of the conditions so provided, before he should be ballotted for, and have the privilege of withdrawing his name, should such provisions prove unsatisfactory.

This accounts for the fact that but one was elected during the present session, it being impracticable to give such notice to the others during the session.

Although it is to be regretted that the application of any worthy man should not receive prompt attention and action, the Society could not well, under the circumstances, have done otherwise than adopt the course they did, considering the vital importance of the object to be gained. Although under the constitution, as revised, it will be far more difficult to obtain membership than it has hitherto been, yet it seems to us that the restrictions are not more than commensurate with the demand; and that the requirements now in force will prove satisfactory to all who would seek membership for noble ends. It cannot be too fully impressed, either upon the minds of the members or upon the profession generally, that the *character of the Society will accord strictly with the character of its members.* It would be idle to expect a good and substantial edifice composed of bad and improper materials.

Let the American Society open the door for the free access of all who may, perhaps for selfish motives, apply for membership, and, although we might soon boast of numbers, yet it would inevitably annihilate that distinction which membership should confer, and necessarily destroy that influence without which this association would become worthless to the profession.

The other topic alluded to, as occupying a large share of this session, was the subject of amalgams, as presented by the recording secretary's report.

Although, for reasons which appear in this report, this subject was not fully disposed of, yet it is plain from the minutes that some advance has been made in the accomplishment of the object which the Society had in view in ordering the circular issued by the recording secretary. The discussion of this subject, so far as its *merits or demerits* are concerned, may be regarded as fully closed, nor was *this* discussion attempted to be revived during the present session. The whole time which it occupied was devoted to it as connected with the report above alluded to. Although this report shows that the circular protest had failed to accomplish the *full* end of its mission, and that the resolutions which accompanied it, in their practical results, had not done equal and exact justice to all, yet the general aspect of the case, as shown by said report, must be regarded as favorable; and is, in fact, a strong guarantee for the future triumph of the Society over this species of quackery.

This report shows, that of the *one hundred and thirty-four* members composing the society at the time the proscriptive resolutions were passed, *only three,* in their response to the circular, *refused* to sign the protest, and *but one* of these based his refusal on an approval of the practice under any circumstances.

But there is another feature in the case, much less gratifying. This report shows, also, that *fifty three* failed *directly* to respond to the circular, and hence failed to comply with the requirements of the Society upon this subject. When this list was presented to the Society, it was found to contain many, nay, to be composed *mainly* of those whose opinions and practice, as was well known to the Society, were wholly averse to the use of amalgam, in *any* case, for dental purposes. But the resolutions made no provision for this unpleasant exigency, and hence, till some provision could be made for them, they could not be regarded as members in full standing. This state of things called for prompt and judicious action, and it is truly gratifying to record the generosity of feeling exhibited on that occasion, on the part of all concerned, and it declares, that some motive more pure and noble than mere selfishness prompted the deliberation. No sooner was this feature in the case presented, than those who had been most zealous in the support and passage of the expulsatory resolutions, were as earnestly engaged in making such provisions as that all who were disposed to act consistently might be restored. To this end, the *third* and *fourth* resolutions accompanying the protest were *unanimously* repealed, and a new test adopted by which a similar exigency could not again occur. To the *fifty-three* who were reported as unheard from, the Society directed the recording secretary to send a second circular, to which each is requested to respond, and the information so derived is to be acted upon at the next annual meeting. It seems to us that this plan takes away every objectionable feature complained of in the plan adopted in the resolutions of last year, and will be equally efficient in securing the object in view. This subject has been one exceedingly trying to the Society, and has elicited feeling and discussion not altogether profitable or pleasant. Supposed personalities are sure to creep into every contention, involving personal interest or reputation, and it is of course impossible to condemn a practice in general, without involving the practice of those participating in it. This remark, as applied to ourself, has, to our knowledge, but a single application, where our remarks, as connected with the discussions upon the subject of amalgams have been demurred to, on the score of dealing in improper personalities.

"It was asserted by us," (among other things,) "in the Journal, that Dr. E. Baker took part in a certain measure against the Mallans; and as he still avers to the Society that such was not the case, and as the witnesses, on whose evidence rested this charge, refused to testify to the Society upon this subject, we therefore most cheerfully withdraw it, as based upon the testimony above alluded to.

During the present meeting, too, very important measures were proposed for future action, which, if carried out efficiently, will greatly benefit the Society and the profession at large.

The one pertaining to the adoption of some uniform system by the Society, by which each member should annually report to the Society all the important cases which had been connected with his practice, or had fallen under his observation during the year; the other was a proposition for the Society to adopt *a code of ethics*, which would serve to define and regulate many points which can never be anticipated in any constitution or code of by-laws.

The former of these measures was proposed by Dr. C. O. Cone, and was ably advocated by him. It is a proposition which commends itself to the good sense of all. One of the main objects of the Society is to concentrate and again diffuse practical information; and the adoption of some well framed and uniform system, in the form of a "tabular sheet," upon which should be arranged the different subjects upon which facts are to be collected, would tend greatly to the accomplishment of this desirable object.

We hope to see this measure efficiently carried into operation, and the names attached to the committee upon this subject may be regarded as a sufficient guarantee that such will be the case.

The other proposition, which was to adopt a system or code of ethics, we deem also of great importance. The history of almost every voluntary association proves that new and unexpected exigencies are constantly occurring, calling for some revision or amendment of its constitution or by-laws, and we venture to assert, that if these cases be examined carefully, it will be found that, in nine out of every ten, the exigency could have been provided for, in a judicious code of ethics. For example, the constitution of the American Society of Dental Surgeons declares, that a member may be expelled for malpractice. But who is to decide what constitutes malpractice? The constitution does not define it, nor could one be well framed, without being cumbersome, which would. Again, this constitution, after enumerating several grounds for expulsion, says that members may be expelled "for other sufficient cause." Now who will define the meaning of *this* phrase.

That the Society have a *right* to define it, is most clear; but this, as we have seen in the case of the amalgam subject, may involve a great deal of perplexity and discussion.

Other cases might be mentioned, exhibiting a strong demand for more definiteness, in order to insure ease and uniform satisfaction in the transaction of business. While it would be most difficult to frame a constitution (which should always be as short and explicit as possible) which would meet all these points, it would be easy to reach them through a well drafted code of ethics, and the adoption of these regulations or principles of the Society, thus embodied, might be enjoined on every member *by* the constitution. It seems to us to be the only way by which we shall be freed from the necessity of *annually* changing some feature in the constitution or by-laws, with the view of meeting some new and unexpected exigency. It is to be hoped that every member will direct his attention to this subject, that such a code may be adopted as will prove satisfactory to all.

The state *Medical Society* of New York have an excellent code, which is adopted by every county medical society in said state, and much instruction may be derived from it. Other states have doubtless adopted a similar suit of regulations. I should esteem it a favor, if the members residing in the different states would forward me, at an early day, the code adopted in the state in which they respectively reside.

In conclusion, we will say a word relative to the adjournment or place of holding the next meeting. It has hitherto been regarded expedient to meet in some one of the principal cities, with a view to accommodate the greatest number of the members. But as our annual meetings are held in the hottest season of the year, this is objectionable, particularly to country members, nor can it be much less so to those residing in the cities.

There is another objection to its being held in the large cities, or any place where a large portion of the members reside. It is a very hard matter for a dentist in full practice to shake off the duties of his profession, while he is known to be in the immediate vicinity of his office. Hence we have uniformly secured the smallest attendance, and the worst attention has been given to the business of the Society, in the city where the greatest number of members reside.

The next annual meeting is to be held at *Saratoga Springs,* one of the most delightful spots on the globe during the warm season, and perhaps no location is better calculated to secure a full attendance at this season of the year. We can here unite pleasure with business, and should any member find himself in *bad humor,* we have no doubt that a free use of this water, "long celebrated for bad humors," will "set all to rights."—*Syracuse Ed.*

